# Supplementary material for: NHC-gold compounds mediate immune suppression through induction of AHR-TGFβ1 signalling in vitro and in scurfy mice
Source: Commun Biol. 2020 Jan 3;3:10. doi: 10.1038/s42003-019-0716-8 (PMC6941985; doi:10.1038/s42003-019-0716-8)
Supplement: Supplementary file 1 — Supplementary Information [file 42003_2019_716_MOESM1_ESM.docx]

**Supplementary Figures**

**
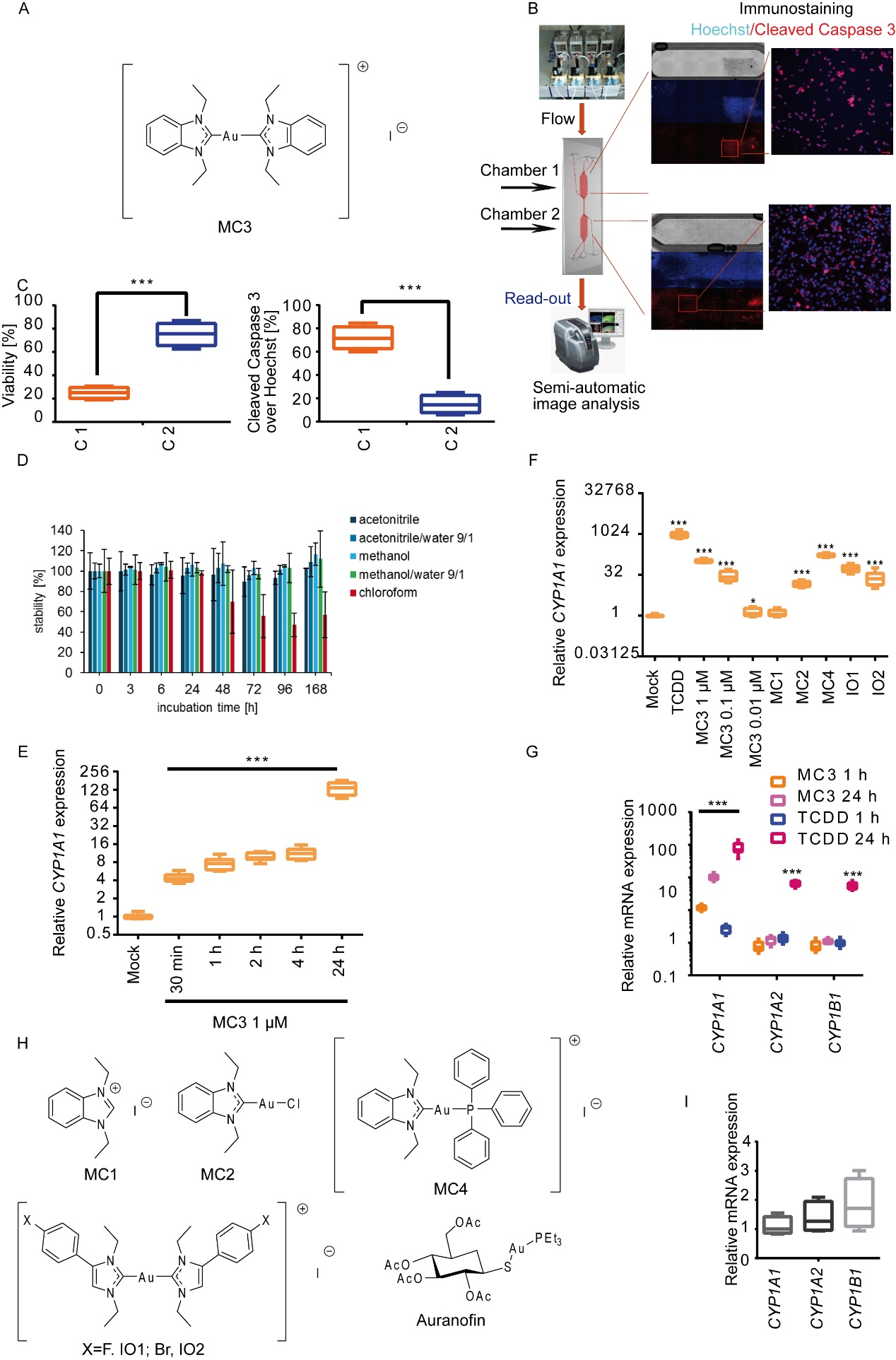
**

**Supplementary Figure 1:** Gold compounds induce CYP1s expression. **A)** Chemical structure of MC3, [di-(1,3-diethylbenzylimidazol-2-ylidene)]gold(I) iodide (n=4). **B)** Schematic workflow of organ-on-chips for monitoring the toxicity of hepatic cells-mediated chemical metabolites. Hoechst dye (blue) and cleaved Caspase 3 (red) were used for evaluation of cell viability and toxicity of chemicals. Scale bar: 40 µm. **C)** *Left:* Viability is calculated by counting the number of Hoechst positive cells treated with MC3 over mock control (DMF). C1 and C2: Chamber 1 and 2. *Right:* Percentage of cleaved Caspase 3 positive cells treated with MC3 over Hoechst positive cells. *Lower right:* Percentage of cleaved Caspase 3 positive cells in various cell combinations. >: flow direction. D) Stability of MC3 in various solvents at 37°C over a period of 168 h (n=2). E) Time-dependent CYP1A1 expression in HepG2 cells treated with MC3 1 µM. F) NHC gold compounds induce CYP1A1 expression. 1 µM of MC1, MC2, MC4, IO1 and IO2 was used. G) Comparison of expression of AHR-associated CYP1 family genes in HepG2 cells upon MC3 1 µM or TCDD 10 nM treatment for 1 h and 24 h (n=9). H) Chemical structures of MC1, MC3, MC4, IO1 and IO2 and auranofin. I) Expression of CYP1s upon auranofin treatment in HepG2 cells (n=4). One-way ANOVA t-test was performed. *: p<0.05, **: p<0.01, ***: p<0.001. Lower and upper ends of the bars indicate the minimum and maximum values, respectively, and the centre represents the median. Error bars ± SD. The source data for 1G and 1I are provided as Supplementary Data 1.


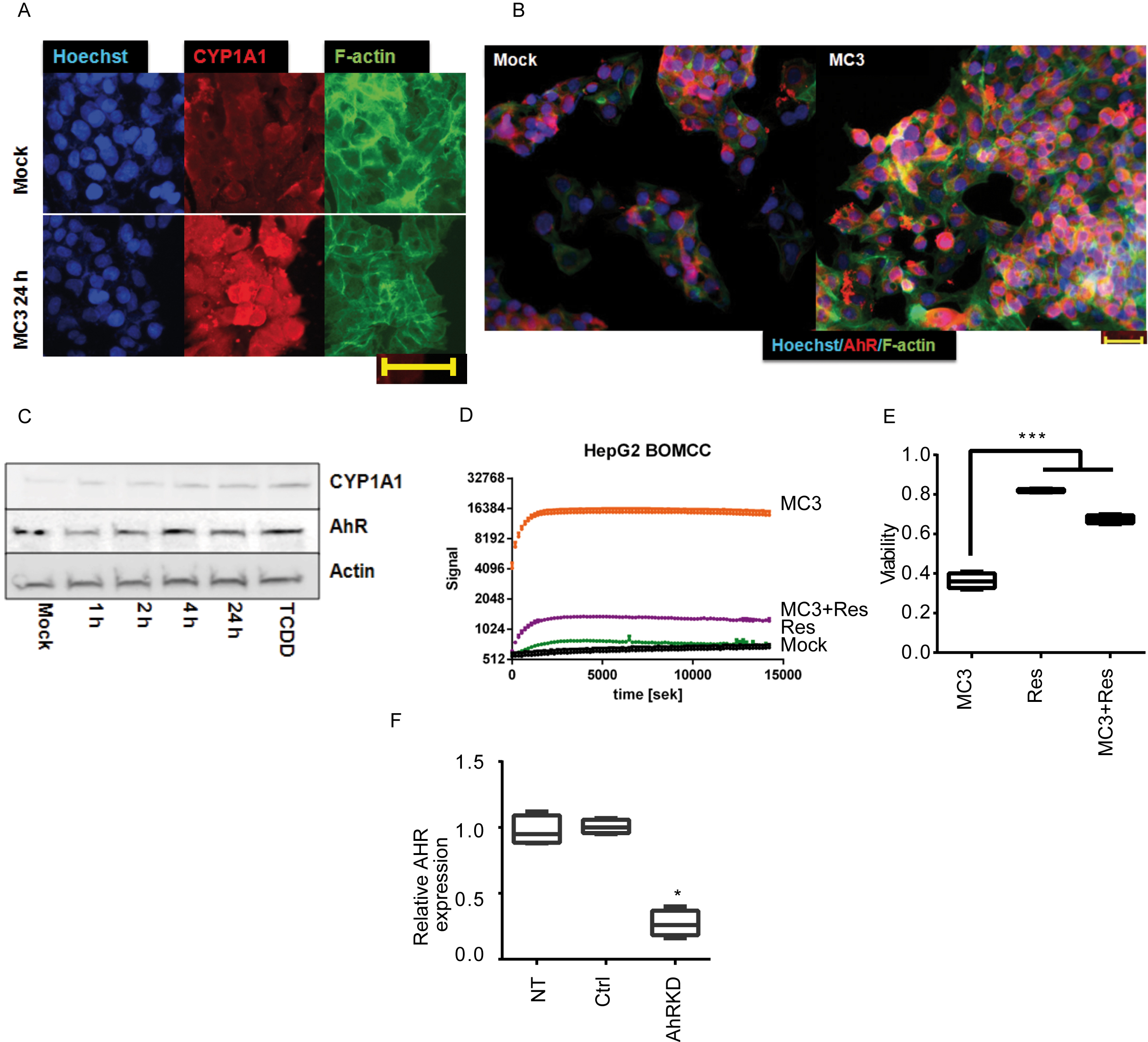


**Supplementary Figure 2:** Gold compounds are potent AHR ligands. A) Induction of CYP1A1 by MC3 (0.5 µM for 24 h) using CYP1A1 specific antibody (red). Scale bar: 40 µm. B) Merged image of nuclear accumulation of AhR upon MC3 0.5 µM treatment for 24 h visualized by labelling with an AHR specific antibody (red) in immunocytochemistry. Scale bar: 40 µm. C) Time-dependent induction of CYP1A1 in HepG2 cells analyzed by immunoblotting. Actin was used as loading control. HepG2 cells treated with TCDD at 10 nM for 24 h was used as positive control and DMF (0.01%) as mock treatment. D) Resveratrol compromises MC3-induced CYP1A1 activity. CYP1A1 enzyme activity was measured by Vividye BOMCC as previously described.^1^ E) Resveratrol antagonizes the toxic effect of MC3 in HepG2 cells detected by MTT assay (n=4). F) Knockdown effect of CRISPR/CAS9 against AhR in HepG2 cells analyzed by qRT-PCR. NT: HepG2 wt; Ctrl: CRISPR/CAS9 non-targeting control; AhR KD: HepG2 CRISPR/CAS9 knockdwon. In A and B Alexa Fluor 488 phalloidin (Green) was used to detect filamentous actin (F-actin). Hoechst dye was used for nucleic staining. Scale bar: 40 µm.One-way ANOVA t-test was performed. *:p<0.05, **: p<0.01, ***: p<0.001. Lower and upper ends of the bars indicate the minimum and maximum values, respectively, and centre represents the median. Error bars ± SD. The source data for 2E is provided as Supplementary Data 1.


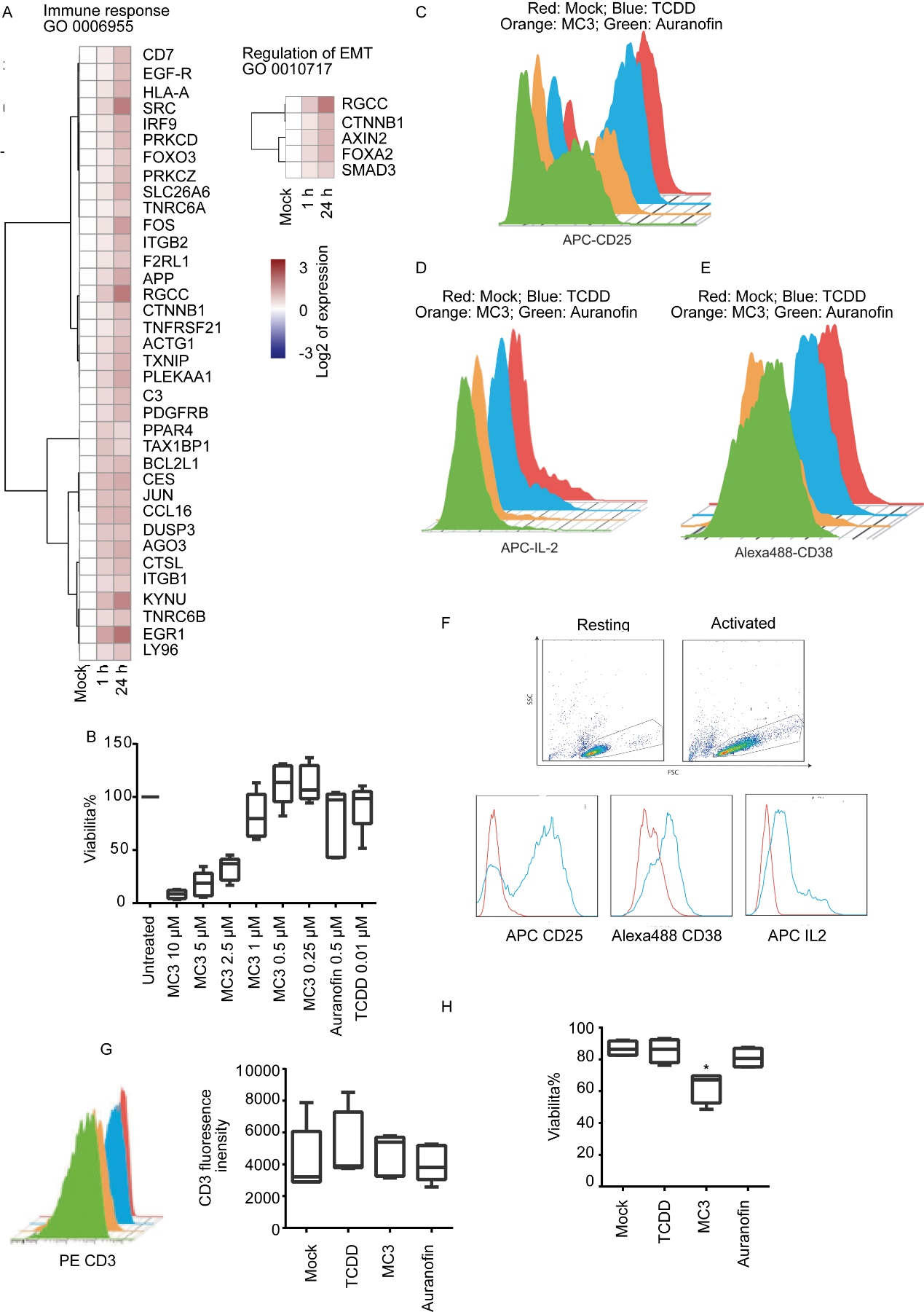


**Supplementary Figure 3:** Immunosuppressive effect of MC3 in human primary T-cells. A) Expression profiles of gene sets annotated with Gene Ontology categories related to selected cellular responses in MC3-treated HepG2 profiled at 1h (HepG2_MC3_1h) and 24h (HepG2_MC3_24h) after treatment. The categories include *‘Immune response’, and ‘Epithelial to mesenchymal transition’.* The selected genes belong to two (out of two) clusters of statistically significant gene profiles detected using STEM (https://www.ncbi.nlm.nih.gov/pubmed/16597342) at significance level of 0.05. The four categories were statistically significantly enriched in one of the clusters at significance level of 0.05. Note that the heatmaps visualize the fold changes on log2 scale, with red color corresponding to up-regulation, blue color corresponding to down-regulation, and the color intensity is corresponding to the strength of dysregulation. B) Viability of resting primary CD4^+^ T cells as measured by MTT assay. Cells were left untreated or incubated overnight with MC3, auranofin or TCDD at the concentrations shown. Absorbance values were normalized to controls following subtraction of the blank (medium only). n=5. C)-E): Histogram of C) CD25, D) IL-2 and E) CD38 intensity upon treatment obtained from FACS analysis. F) Illustration of gating population for FACS analysis in primary CD4+ T cells. G) CD3 expression levels in activated primary CD4^+^ T-cells. Resting CD4^+^ T-cells were left untreated or incubated overnight with 0.5 µM MC3, 0.5 µM auranofin or 10 nM TCDD. Cells were then activated through CD3-CD28 stimulation and CD3 levels were measured by flow cytometry (n=5). H) Viability of activated CD4^+^ T cells as measured by live/dead stain. Resting cells were left untreated or incubated overnight with MC3, auranofin or TCDD. Cells were then washed and activated with αCD3-CD28 beads for 24 hours and assayed for viability (n=4). Data were analyzed by One-way ANOVA followed by Dunnett's multiple comparisons post-test. One-way ANOVA t-test was performed. *:p<0.05, **: p<0.01, ***: p<0.001. Lower and upper ends of the bars indicate the minimum and maximum values, respectively, and the centre represents the median. Error bars ± SD. The source data for 3B, 3G, and 3H are provided as Supplementary Data 1.


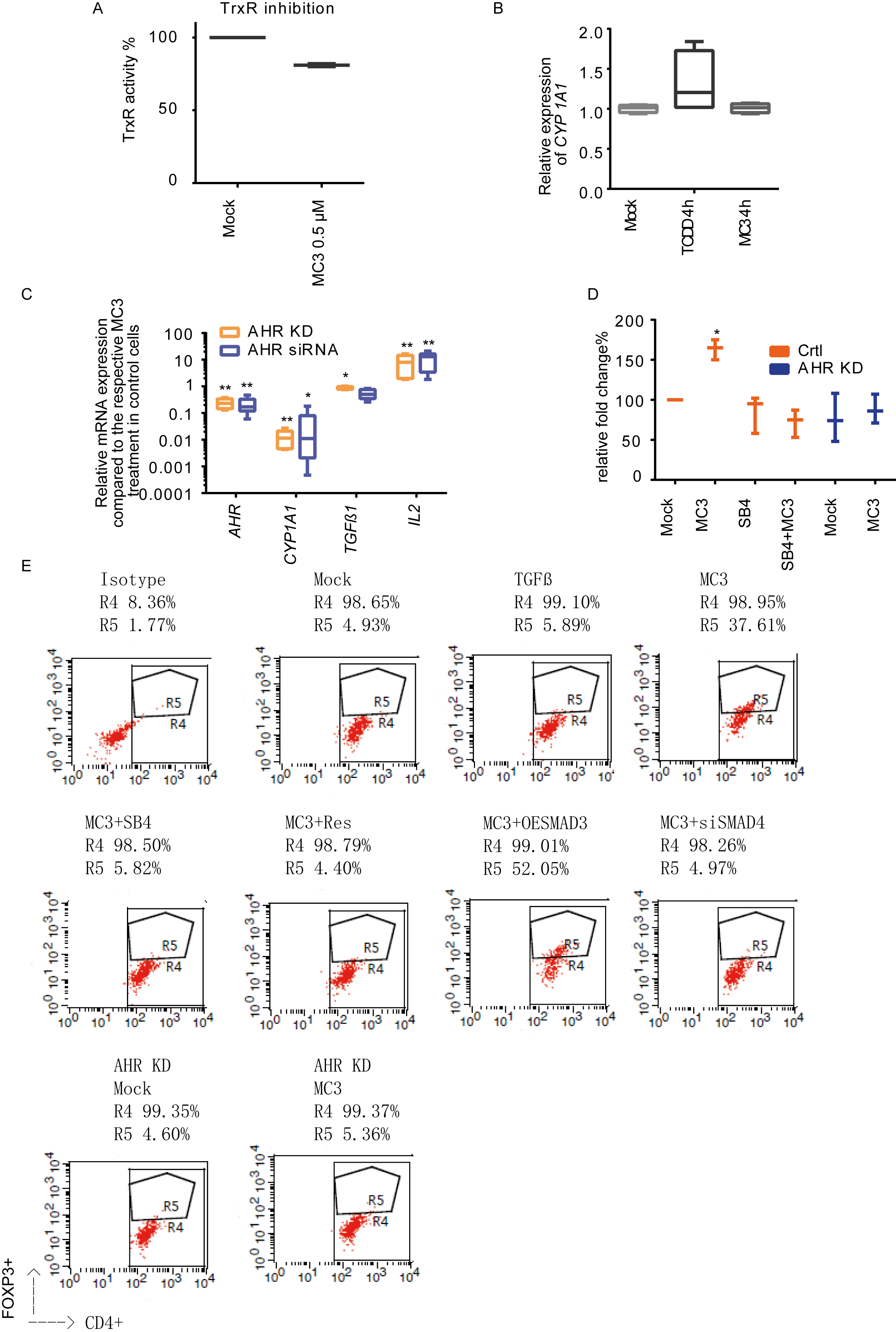


**Supplementary Figure 4:** Immunosuppressive effect of MC3 *in vitro*. A) Inhibition of TrxR by MC3 in *in vitro* enzymatic assay (n=3). B) CYP1A1 expression in Jurkat cells with or without treatment (n=4). C) Quantification of signalling intensity from immunoblotting in Fig. 3D (n=6). D) FACS analysis of CD4^+^FOXP3^+^ SupT1 cells upon treatments as indicated (n=3). Gate: R4 is defined as CD4+ cells; R5 is defined as CD4^+^FOXP3^+^ cells One-way ANOVA t-test was performed. *:p<0.05, **: p<0.01, ***: p<0.001. Lower and upper ends of the bars indicate the minimum and maximum values, respectively, and the centre represents the median. Error bars ± SD. The source data for 4A, 4B, 4C and 4D are provided as Supplementary Data 1.


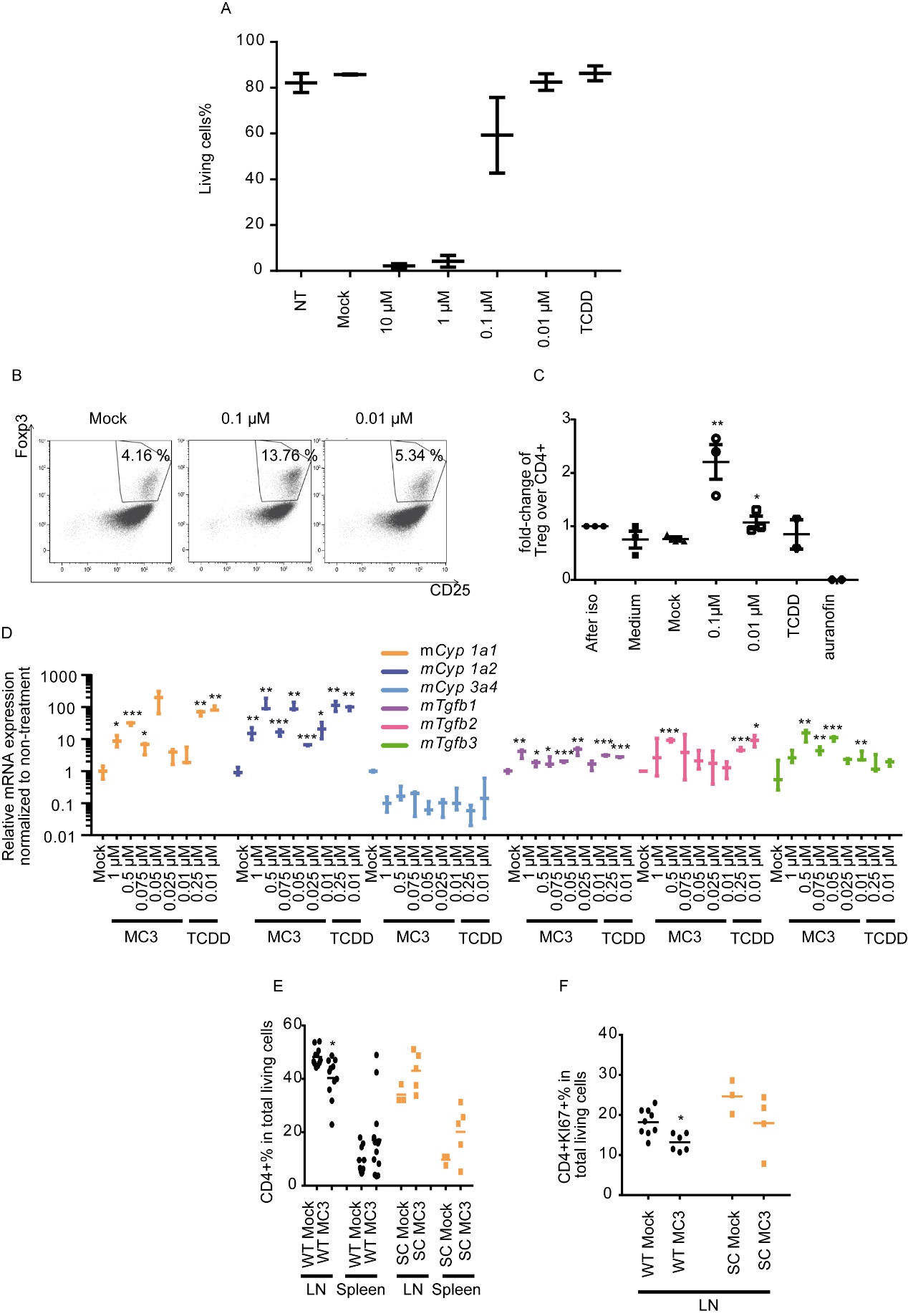


**Supplementary Figure 5:** Immunosuppressive effect of MC3 *in vivo*. A) The percentage of living cells upon treatments (n=2). B) FACS analysis of CD4^+^CD25^+^FOXP3^+^ cells in murine lymph nodes. Gate: CD25^+^FOXP3^+^ in CD4^+^ T-cells. C) Percentage of Treg over CD4^+^ after various treatments indicated (n=3). D) Expression of *Cyp1a1, 1a2, 3a4* and *Tgfß1/2/3* in mouse CD4^+^ T-cells upon treatments (n=3). E) Comparison of CD4^+^ cells in lymph node (LN) and spleen (n≥3). F) The percentage of CD4^+^KI67^+^ in lymph node (n≥3). One-way ANOVA t-test was performed. *:p<0.05, **: p<0.01, ***: p<0.001. Lower and upper ends of the bars indicate the minimum and maximum values, respectively, and the centre represents the median. Error bars ± SD. The source data for 5A, 5B, 5C, 5D, 5E and 5E are provided as Supplementary Data 1.


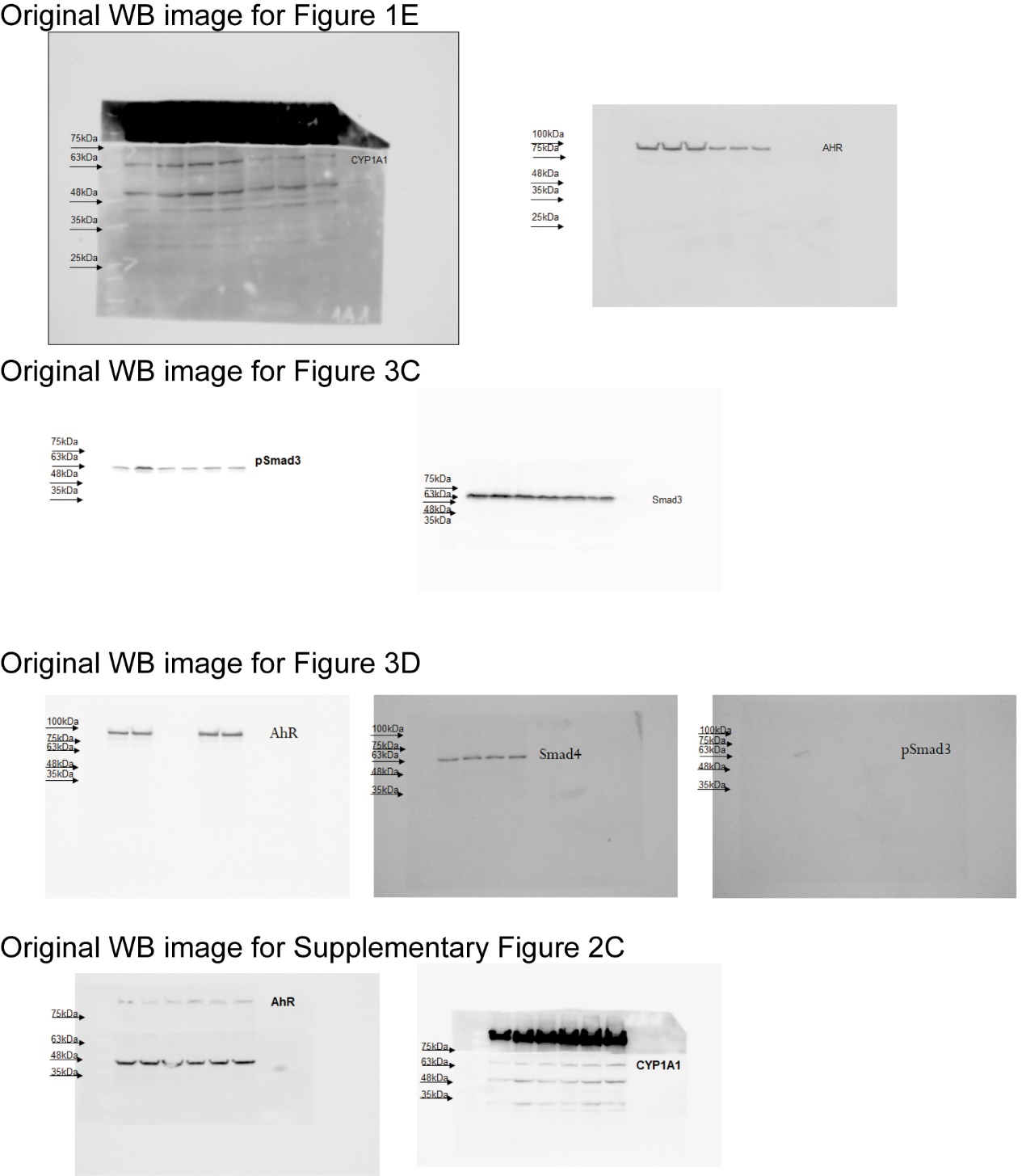


**Supplementary Figure 6:** original WB images for figure 1E, 3C, 3D and supplementary figure 2C.

**Supplementary Notes, Discussion and Methods**

**Chemistry**

Scheme: Synthesis of MC1, MC2 and MC3. a) ACN, C_2_H_5_I, K_2_CO_3_, 8 h, reflux; b) Ag_2_O, DCM, 4 h, RT; c) SMe_2_AuCl, 24 h, RT; d) MC1, DCM/MeOH (1/9), K_2_CO_3_, 4 h, RT.

SI. 6: ^1^H-NMR spectra of MC1, MC2 and MC3 (400 MHz, CDCl_3_).

MC3 was prepared by an improved synthesis procedure based on a recently reported method.^2,3^ In general, MC3 is prepared in a three step procedure starting from benzimidazole, which is alkylated in a first step using ethyliodide. The purified diethylbenzimidaziolium iodide is then reacted with silver oxide to form a silver carbene intermediate, which is not isolated and treated in a one-pot reaction with chloro-dimethylsulfidegold (I). A transmetallation takes place resulting in the gold NHC complex MC2. The second NHC ligand is introduced by reaction of MC2 with diethylbenzimidazolium iodide resulting in the bis-NHC compound MC3. The high purity of all compounds was confirmed by elemental analysis (maximum difference between theoretical and experimental values: 0.4 %). The yields of the respective compounds could be substantially improved by a stepwise optimization of reaction conditions (time, solvents) as well as work-up and isolation procedures. The yields from repeated experiments were as follows: 98% for MC1, 91-96 % for MC2 and 76-96% for MC3.

**1,3-Diethylbenzimidazolium iodide (MC1)^2^**

To a solution of benzimidazole (1.00 g; 8.465 mmol; 1 eq) in acetonitrile (10 mL) potassiumcarbonate (1.17 g; 8.465 mmol, 1 eq) was added followed by ethyliodide (2.00 mL; 25.39 mmol; 3 eq). The mixture was stirred under reflux conditions for 8 h. After cooling down, the solvent was removed in *vacuo*. The residue was resuspended in dichloromethane and extracted with destilled water. The combined organic phases were dried over Na_2_SO_4_ and concentrated to give MC1 as a white solid (98.3 %; 2.513 g). **^1^H-NMR** (400 MHz, CDCl_3_) δ (ppm) = 11.13 (s, 1H, N*H*C), 7.74 (dd, *J* = 6.3, 3.2 Hz, 2H, ArH_5/6_), 7.68 (dd, *J* = 5.9, 3.4 Hz, 2H, ArH_4/7_), 4.70 (q, *J* = 7.4 Hz, 4H, CH_2_), 1.77 (t, *J* = 7.4 Hz, 6H, CH_3_); **^13^C-NMR** (101 MHz, CDCl3) δ (ppm) = 141.53 (C_3_, NHC), 131.34 (C_4_, ArC_3a/7a_), 127.42 (C_3_, ArC_4,7_), 113.20 (C_3_, ArC_5/6_), 43.19 (C_2_, CH_2_), 15.05 (C_1_, CH_3_). Elemental analysis for C_11_H_15_N_2_I (% calcd/found): C (43.73/43.81), H (5.00/4.89), N (9.27/9.09).

**Chlorido-(1,3-diethylbenzimidazol- 2-ylidene)gold(I) (MC2)^2^**

MC1 (0.5 g, 1.655 mmol, 2 eq) was treated with di-silveroxide (0.192 g, 0.827 mmol, 1 eq) under vigorous stirring in dichloromethane. After 4 h chlorodimethylsulfidegold(I) (0.487 g, 1.655 mmol, 2 eq) was added and the reaction was stirred for another 24 h. The obtained suspension was filtered over Celite, and the solvent was evaporated under reduced pressure. The residue was resuspended in tetrahydrofurane and hexane was added until the appearance of a precipitate. The white crystalline solid, was isolated by filtration (0.646 g, 1.589 mmol, 96 %). **^1^H-NMR** (600 MHz, CDCl_3_) δ (ppm) = 7.48 (dd, *J* = 6.5, 3.4 Hz, 2H, ArH_5/6_), 7.43 (dd, *J* = 5.8, 3.4 Hz, 2H, ArH_4/7_), 4.54 (q, *J* = 7.3 Hz, 4H, CH_2_), 1.53 (t, *J* = 7.3 Hz, 6H, CH_3_). **^13^C-NMR** (151 MHz, CDCl_3_) δ (ppm) = 177.36 (C_4_, NHC), 132.88 (C_4_, ArC_3a/7a_), 124.55 (C_3_, ArC_4/7_), 111.50 (C_3_, ArC_5/6_), 44.08 (C_2_, CH_2_), 15.61 (2 C_1_, CH_3_). Elemental analysis for C_11_H_14_AuClN_2_ (% calcd/found): C (32.49/32.42), H (3.47/3.29), N (6.89/6.78).

**[Di-(1,3-diethylbenzimidazol-2-ylidene)]gold(I) iodide (MC3)^3^**

A solution of diethylbenzimidazolium iodide (0.1 g, 0.331 mmol, 1 eq) in 10 mL of dichloromethane/methanol (1/9) was treated with MC2 (0.135 g, 0.331 mmol, 1 eq) and potassiumcarbonate (0.046 g, 0.331 mmol, 1 eq) and stirred at room temperature for 4 h. The solvent of the resulting suspension was removed under reduced pressure, and the residue was resuspended in dichloromethane, and hexane was added until the appearance of a precipitate. The solid, a white powder, was isolated by filtration and washed with hexane (0.170 g, 0.253 mmol, 76.4%). **^1^H-NMR** (600 MHz, CDCl_3_)
δ (ppm) = 7.58 (dd, *J* = 9.1, 3.0 Hz, 4H, ArH_5/6_), 7.47 (dd, *J* = 9.2, 2.9 Hz, 4H, ArH_4/7_), 4.72 (q, *J* = 7.3 Hz, 8H, CH_2_), 1.69 (t, *J* = 7.4 Hz, 12H, CH_3_). **^13^C-NMR** (151 MHz, CDCl_3_) δ (ppm) = 190.42 (C_4_, NHC), 133.24 (C_4_, ArC_3a/7a_), 124.78 (C_3_, ArC_4/7_), 111.69 (C_3_, ArC_5/6_), 44.37 (C_2_, CH_2_), 16.20 (C_1_, CH_3_). Elemental analysis for C_22_H_28_AuN_4_I (% calcd/found): C (39.30/39.70), H (4.20/4.29), N (8.33/8.19).

**Stability of MC3 in solution (HPLC-MS studies)**

***Stability measurement***

MC3 was dissolved (1.0 mM) in acetonitrile, acetonitrile/water (v/v: 9/1), methanol, methanol/water (v/v: 9/1) and chloroform. All samples were filtered using a Captiva Econo-Filter PTFE 13m 0.2µm (Agilent) and incubated at 37°C in an incubator. After the indicated periods, aliquots were analysed by high performance liquid chromatography-mass spectrometry (HPLC-MS) by using an Agilent 1620 apparatus equipped with a single quadrupole mass spectrometer (Agilent 6120B); The instrumental setup and chromatographic conditions were as follows: injection volume: 3.0µL; flow rate: 0.6 mL/min; sampler temperature: 37°C, column temperature: 40°C; mobile phase: eluent A: methanol, eluent B: ammonium formiate buffer pH 7.0 with 0.02% formic acid, with operation in gradient mode (0 min: 5% A / 95% B, 1 min: 95% A / 5% B, 4 min: 95% A / 5% B, 4.5 min: 5% A / 95% B, run time: 10 min); stationary phase: ZORBAX Rapid Resolution HT SB-C18 (2.1 x 50 mm, particle size: 1.8 μm); detection by MS or UV spectroscopy; MS: electrospray ionisation (ESI) source (drying gas temp.: 350 °C, flow 12 L/min, nebulizer pressure: 35 psi, capillary voltage: 3000 V, gain: 4, fragmentor voltage: 70 V; UV spectra were measured at 200, 240, 250 and 290 nm. For calibration a dilution series of MC3 in acetonitrile (concentration range: 2.0 mM - 50µM) was freshly prepared daily from a stock solution of 10 mM. The correlation coefficients (r^2^) of the linear calibration function were >0.995. The final quantitative results are presented as percentage of the initial amount of MC3 dissolved in the respective solvent and were obtained in two independent experiments.

***Stability - Results***

The stability of MC3 in solution was studied by HPLC-MS. For this purpose, MC3 was dissolved in acetonitrile, acetonitrile/water 9/1, methanol, methanol/water 9/1 or chloroform, respectively, and incubated at 37°C over a period of 168 h (see figure SI7). HPLC-MS analysis of the acetonitrile, methanol solutions and their mixtures with water showed that MC3 was stable without detection of decomposition products. In the most apolar solvent chloroform, however, MC3 levels were decreased after exposure for more than 24 h. Still, the levels of intact MC3 remained in the 50% range up to 168 h of incubation. Two additional peaks could be observed in this case with mass signals of m/z 175.1 and m/z 191.2. The former one could be clearly identified as the cationic benzimidazolium MC1 (figures SI8, SI9). For the latter one the structure 1,3-diethyl-2,3-dihydro-1H-benzoimidazol-2-olate can be suggested based on literature research. Ref: Pozharskii, A.F., Kuz'menko, V.V., Kashparov, I.S. et al. *Chem. Heterocycl. Compd*., 1976, (12), 304-311). Taken together, MC3 shows an exceptional chemical stability in aqueous and polar environment at physiological temperature over several days, however, it experiences some decomposition after extended periods (> 24 h) in highly apolar medium.


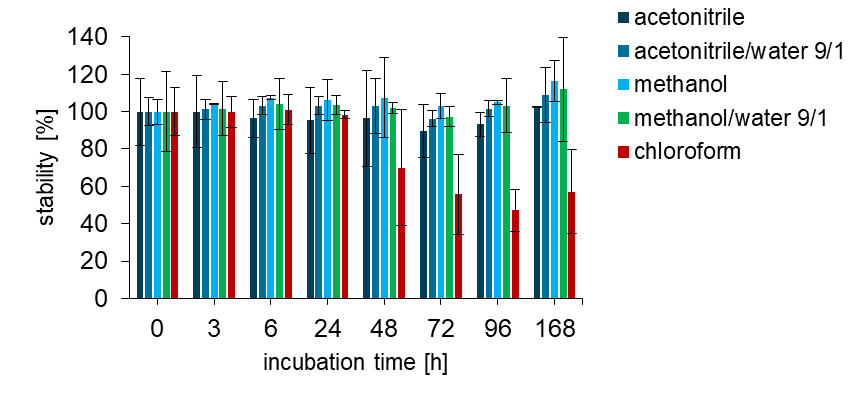


SI. 7: Stability of MC3 in various solvents at 37°C over a period of 168 h (n=2).


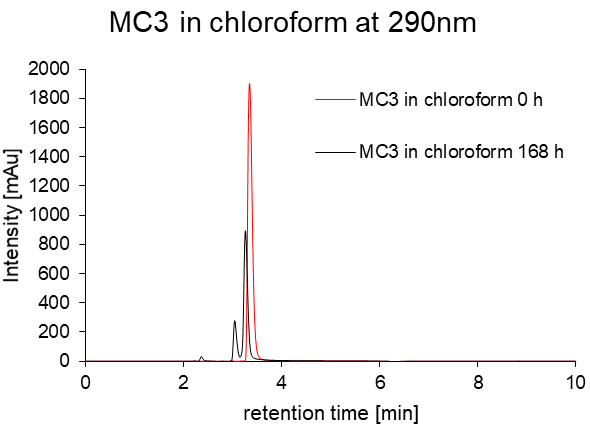


m/z 175.1

m/z 191.2

m/z 545.2


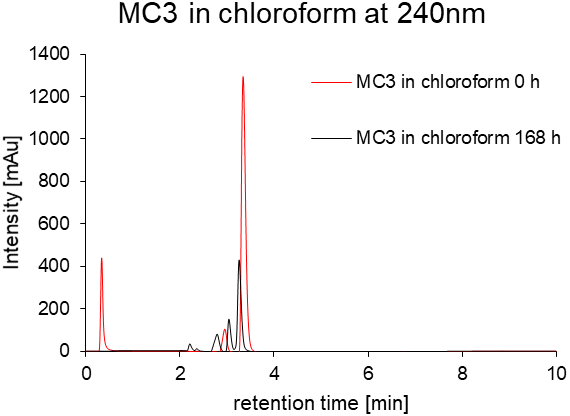


m/z 175.1

CHCl_3_

m/z 191.2

m/z 545.2

SI. 8: Chromatograms of MC3 after 0 and 168 h in chloroform at 37 °C.

**MC1**


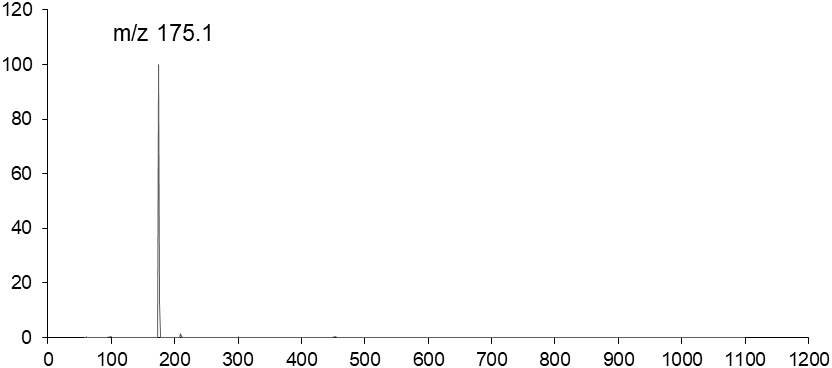


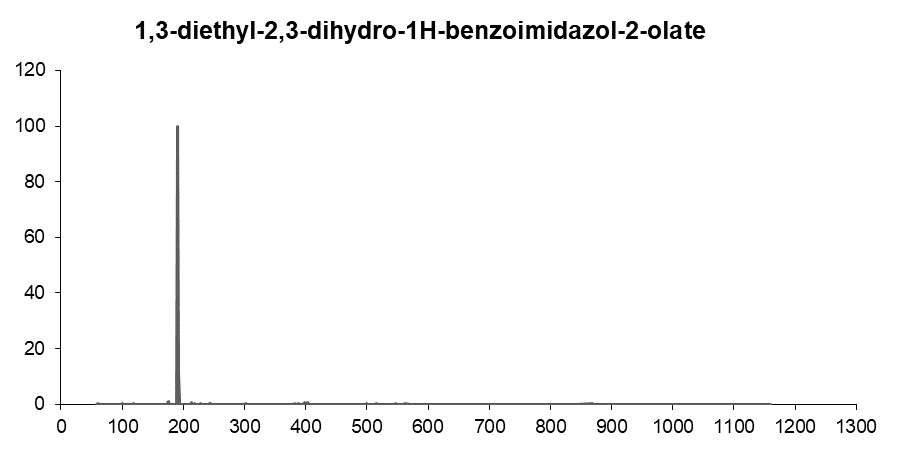


m/z 191.2

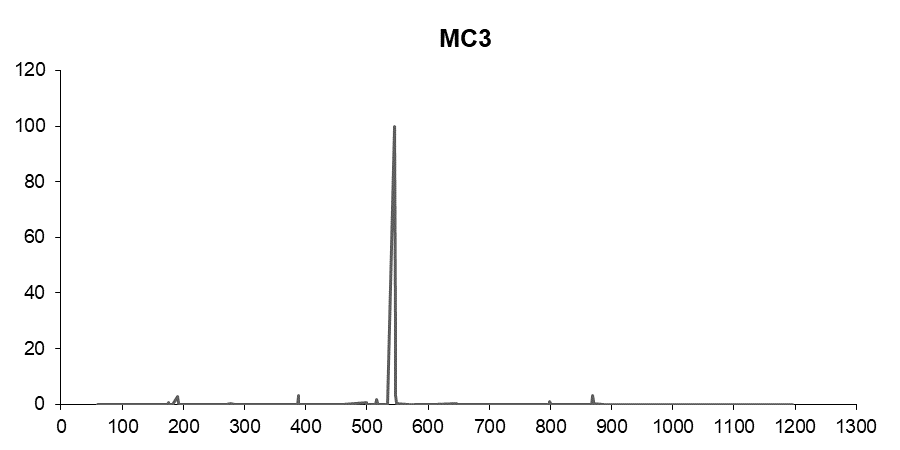


m/z 545.2

SI. 9: Mass and UV spectra of the individual peaks of SI. 8.

Supplementary Table 1: Primer sequences of genes

| **Gene** | **Forward** | **Reverse** | NCBI accession |
| --- | --- | --- | --- |
| CYP3A5 | cagatcccgacgtgatcaga | ttttccgctggtgaaggttg | NM_000777.4 |
| CYP2C8 | gaggaccgtgttcaagagga | gcagatcacattgcagggag | NM_000770.3 |
| CYP2A6 | aacaggcctttcagttgctg | gcatgcggatgagaaaggag | NM_000762.5 |
| CYP11A1 | ttccgctttgcctttgagtc | tggcatcaatgaatcgctgg | NM_000781.2 |
| CYP11B1 | agcatcagtgaacatcccca | agagagtagaggaacacgcg | NM_000497.3 |
| CYP17A1 | ccgcacaccaactatcagtg | cccttgtccacagcaaactc | NM_000102.3 |
| CYP2J2 | ggagaacggacagccttttg | agctggcatgtctttgaagc | NM_000775.3 |
| CYP4A11 | tatggggaggcaaagttcgt | gtcgatgctggaaccatgtc | NM_000778.3 |
| CYP4F2 | gctgagcaaggatgaagacg | ttaggctcacggtccttcag | NM_001082.4 |
| CYP24A1 | acgggcagaagatttgagga | cccagaactgttgccttgtc | NM_000782.4 |
| CYP19A1 | acatctggacaggttggagg | ttgatgaggagagcttgcca | NM_000103.3 |
| CYP1A1 | cttccgacactcttccttcg | ggttgatctgccactggttt | NM_000499.3 |
| CYP1A2 | tccagaaggagctggacact | aaggaggagtgtcggaaggt | NM_000761.4 |
| CYP1B1 | aaccgcaacttcagcaactt | gaggataaaggcgtccatca | NM_000104.3 |
| hAhR | ctgcctttcccacaagatgt | agttatcctggcctccgttt | NM_001621.4 |
| hAhRR | agcggagatgaaaatgagga | agttccgattcgcacagact | NM_001242412.1 |
| hARNT | ctagtggccattggcagatt | caatgttgtgtcgggagatg | NM_001197325.1 |
| hARNTL | aataggccgaatgattgctg | tcttcttgcctcctggagaa | NM_001030272.2 |
| hARNTL2 | aatgggggagctagaggcta | cattgtcacatagggcatcg | NM_001248002.1 |
| hARNT2 | atcatctgcaccaacaccaa | ggggacctgggataagtcat | NM_014862.3 |
| hHSP90 | gggcaacacctctacaagga | atcaactgggcaatttctgc | NM_001017963.2 |
| hCYP2E1 | cctacatggatgctgtggtg | tggggatgaggtatcctctg | NM_000773.3 |
| hCYP2D6 | tgatgagaacctgcgcatag | ccctatcacgtcgtcgatct | XM_011529969.1 |
| hCYP2B6 | tgcggaattgttcctcttct | aagcggatctggtatgttgg | NM_000767.4 |
| hCYP3A4 | accgtgacccaaagtactgg | gtttctgggtccacttccaa | NM_001202855.2 |
| hCYP2C9 | ccacatgccctacacagatg | tgcccttgggaatgagatag | NM_000771.3 |
| TGFß1 | gtggaaacccacaacgaaat | cacgtgctgctccactttta | NM_000660.6 |
| TGFß2 | ttgacgtctcagcaatggag | tcgccttctgctcttgtttt | NM_001135599.3 |
| IL-2 | ggatgcaactcctgtcttgc | tgtgagcatcctggtgagtt | NM_000586.3 |
| hKEAP1 | ctcatccagccctgtcttca | ggtacatgacagcaccgttc | NM_012289.3 |
| hNRF2 | ggttgcccacattcccaaat | agcaatgaagactgggctct | NM_001145412.3 |
| hNQO1 | atggaagaaacgcctggaga | tggttgtcagttgggatgga | NM_000903.3 |
| Actin | CTGACTACCTCATGAAGATCCTC | CATTGCCAATGGTGATGACCTG |  |

**Supplementary References**

1 Theobald, J. *et al.* Monitoring cytochrome P450 activity in living hepatocytes by chromogenic substrates in response to drug treatment or during cell maturation. *Arch Toxicol* **92**, 1133-1149, doi:10.1007/s00204-017-2128-1 (2018).

2 Rubbiani, R. *et al.* Benzimidazol-2-ylidene gold(I) complexes are thioredoxin reductase inhibitors with multiple antitumor properties. *J Med Chem* **53**, 8608-8618, doi:10.1021/jm100801e (2010).

3 Rubbiani, R. *et al.* Comparative in vitro evaluation of N-heterocyclic carbene gold(I) complexes of the benzimidazolylidene type. *J Med Chem* **54**, 8646-8657, doi:10.1021/jm201220n (2011).
