# Supplementary material for: NHC-gold compounds mediate immune suppression through induction of AHR-TGFβ1 signalling in vitro and in scurfy mice
Source: Commun Biol. 2020 Jan 3;3:10. doi: 10.1038/s42003-019-0716-8 (PMC6941985; doi:10.1038/s42003-019-0716-8)
Supplement: Supplementary file 2 — Description of Additional Supplementary Files [file 42003_2019_716_MOESM2_ESM.docx]

Descriptions of additional supplementary file

The supplementary file contains the source data underlying the graphs and charts presented in the article, including Figures 1B, 1C, 1D, 2B, 2C, 2D, 2E, 3A, 3B, 3E, 3F, 3G, 4A, 4B, 4C, 4D, 4F, 4G, 4H, 5A, 5B, 5C and 5E, as well as supplementary figures 1G, 1I, 2E,3B, 3G, 3H, 4A, 4B, 4C, 4D, 5A, 5C, 5D, 5E and 5F.
