# Supplementary material for: NHC-gold compounds mediate immune suppression through induction of AHR-TGFβ1 signalling in vitro and in scurfy mice
Source: Commun Biol. 2020 Jan 3;3:10. doi: 10.1038/s42003-019-0716-8 (PMC6941985; doi:10.1038/s42003-019-0716-8)
Supplement: Supplementary file 4 — Reporting Summary [file 42003_2019_716_MOESM4_ESM.pdf]

nature research

Corresponding author(s): Xinlai Cheng

Last updated by author(s): Nov 13, 2019

Reporting Summary

Nature Research wishes to improve the reproducibility of the work that we publish. This form provides structure for consistency and transparency in reporting. For further information on Nature Research policies, see Authors & Referees and the Editorial Policy Checklist.

Statistics

For all statistical analyses, confirm that the following items are present in the figure legend, table legend, main text, or Methods section.

n/a	Confirmed	
☐	☒	The exact sample size (*n*) for each experimental group/condition, given as a discrete number and unit of measurement
☐	☒	A statement on whether measurements were taken from distinct samples or whether the same sample was measured repeatedly
☐	☒	The statistical test(s) used AND whether they are one- or two-sided<br>*Only common tests should be described solely by name; describe more complex techniques in the Methods section.*
☒	☐	A description of all covariates tested
☐	☒	A description of any assumptions or corrections, such as tests of normality and adjustment for multiple comparisons
☒	☐	A full description of the statistical parameters including central tendency (e.g. means) or other basic estimates (e.g. regression coefficient) AND variation (e.g. standard deviation) or associated estimates of uncertainty (e.g. confidence intervals)
☒	☐	For null hypothesis testing, the test statistic (e.g. *F*, *t*, *r*) with confidence intervals, effect sizes, degrees of freedom and *P* value noted<br>*Give P values as exact values whenever suitable.*
☒	☐	For Bayesian analysis, information on the choice of priors and Markov chain Monte Carlo settings
☒	☐	For hierarchical and complex designs, identification of the appropriate level for tests and full reporting of outcomes
☒	☐	Estimates of effect sizes (e.g. Cohen's *d*, Pearson's *r*), indicating how they were calculated

Our web collection on statistics for biologists contains articles on many of the points above.

Software and code

Policy information about availability of computer code

Data collection	no software was used
Data analysis	Prism, excel

For manuscripts utilizing custom algorithms or software that are central to the research but not yet described in published literature, software must be made available to editors/reviewers. We strongly encourage code deposition in a community repository (e.g. GitHub). See the Nature Research guidelines for submitting code & software for further information.

Data

Policy information about availability of data

All manuscripts must include a data availability statement. This statement should provide the following information, where applicable:

- Accession codes, unique identifiers, or web links for publicly available datasets
- A list of figures that have associated raw data
- A description of any restrictions on data availability

Microarray accesiosn number GSE125146

Field-specific reporting

Please select the one below that is the best fit for your research. If you are not sure, read the appropriate sections before making your selection.

☒ Life sciences ☐ Behavioural & social sciences ☐ Ecological, evolutionary & environmental sciences

For a reference copy of the document with all sections, see nature.com/documents/nr-reporting-summary-flat.pdf

Life sciences study design

All studies must disclose on these points even when the disclosure is negative.

Sample size	at least three independent experiments were performed.
Data exclusions	nothing
Replication	all results are reproducible tested from at least three independent experiments
Randomization	Mice were randomized for the treatment
Blinding	The investigators were blinded to mice experiments

Reporting for specific materials, systems and methods

We require information from authors about some types of materials, experimental systems and methods used in many studies. Here, indicate whether each material, system or method listed is relevant to your study. If you are not sure if a list item applies to your research, read the appropriate section before selecting a response.

Materials & experimental systems

n/a	Involved in the study	
☐	☒	Antibodies
☐	☒	Eukaryotic cell lines
☒	☐	Palaeontology
☐	☒	Animals and other organisms
☐	☒	Human research participants
☒	☐	Clinical data

Methods

n/a	Involved in the study	
☒	☐	ChIP-seq
☐	☒	Flow cytometry
☒	☐	MRI-based neuroimaging

Antibodies

Antibodies used	CD4 invitrogen 12-0041-82, Foxp3 Alexa fluo488 invitrogen 53-4774-42, AhR Biomol AP5533c, Cyp1A1 Biomol F50940, Cleaved Caspase-3 (D175) (5A1E) Cell signaling 9664P,
Validation	Except for the validation provided by companies we also performed qPCR experiments to confirm the regulation of gene expression upon treatments.

Eukaryotic cell lines

Policy information about cell lines

Cell line source(s)	All cell lines were purchased from ATCC
Authentication	Cells freshly isolated from human patients or mice were not authenticated
Mycoplasma contamination	No mycoplasma contamination was confirmed by qPCR
Commonly misidentified lines (See ICLAC register)	No misidentified line was used in this project.

Animals and other organisms

Policy information about studies involving animals; ARRIVE guidelines recommended for reporting animal research

Laboratory animals	Scurfy Mice (B6.Cg-Foxp3sf/Y) were purchased from Jackson Laboratories (Bar Harbor, ME, USA).
Wild animals	C57BL/6 mice were used as wild type mice
Field-collected samples	not applicable
Ethics oversight	All animal experiments were approved with the animal proposal number G202-17 (Regierungspräsidium Karlsruhe, Baden-Württemberg, Germany)

Note that full information on the approval of the study protocol must also be provided in the manuscript.

Human research participants

Policy information about studies involving human research participants

Population characteristics	not applicable
Recruitment	not applicable
Ethics oversight	Primary CD4+ T-cells were isolated from total blood obtained from healthy donors in agreement with the regulations of the Institute for Clinical Transfusion Medicine and Cell Therapy (IKTZ, in Heidelberg).

Note that full information on the approval of the study protocol must also be provided in the manuscript.

Flow Cytometry

Plots

Confirm that:

☒ The axis labels state the marker and fluorochrome used (e.g. CD4-FITC).

☒ The axis scales are clearly visible. Include numbers along axes only for bottom left plot of group (a 'group' is an analysis of identical markers).

☒ All plots are contour plots with outliers or pseudocolor plots.

☒ A numerical value for number of cells or percentage (with statistics) is provided.

Methodology

Sample preparation	cells were trypsinized, washed with PBS and labeled with respective antibodies with or without fixation and permeabilization.
Instrument	Analysis was performed with flow cytometer (Gallios, Beckman Coulter, Sinsheim, Germany).
Software	FACS data analysis was performed using the Kaluza software.
Cell population abundance	Cells were labelled with respective antibodies
Gating strategy	antibody labelled cells were gated

☒ Tick this box to confirm that a figure exemplifying the gating strategy is provided in the Supplementary Information.
